# The risks associated with tourniquet use in lower limb trauma surgery: a systematic review and meta-analysis

**DOI:** 10.1007/s00590-021-02957-7

**Published:** 2021-04-01

**Authors:** Muhamed M. Farhan-Alanie, Fatema Dhaif, Alex Trompeter, Martin Underwood, Joyce Yeung, Nick Parsons, Andy Metcalfe, Peter D. H. Wall

**Affiliations:** 1 Academic Clinical Fellow Specialty Trainee in Trauma and Orthopaedic Surgery, Warwick Medical School & University Hospital Coventry & Warwickshire, Coventry, UK; 2 Consultant Orthopaedic Trauma Surgeon, Reader in Orthopaedic Surgery, St George’s University Hospital NHS Foundation Trust, St George’s University of London, London, UK; 3 Warwick Clinical Trials Unit, University of Warwick, Coventry, CV4 7AL UK; 4 Warwick Medical School, University of Warwick, Coventry, CV4 7AL UK; 5 Consultant Orthopaedic Surgeon and Associate Clinical Professor in Trauma and Orthopaedic Surgery, Warwick Medical School & University Hospital Coventry & Warwickshire, Coventry, UK

**Keywords:** Fracture, Trauma, ORIF, Lower limb, Tourniquet

## Abstract

**Purpose:**

Tourniquet use in lower limb fracture surgery may reduce intra-operative bleeding, improve surgical field of view and reduce length of procedure. However, tourniquets may result in pain and the production of harmful metabolites cause complications or affect functional outcomes. This systematic review aimed to compare outcomes following lower limb fracture surgery performed with or without tourniquet.

**Methods:**

We searched databases for RCTs comparing lower limb fracture surgery performed with versus without tourniquet reporting on outcomes pain, physical function, health-related quality of life, complications, cognitive function, blood loss, length of stay, length of procedure, swelling, time to union, surgical field of view, volume of anaesthetic agent, biochemical markers of inflammation and injury, and electrolyte and acid–base balance. Random-effects meta-analysis was performed. PROSPERO ID CRD42020209310.

**Results:**

Six RCTs enabled inclusion of 552 procedures. Pooled analysis demonstrated that tourniquet use reduced length of procedure by 6 minutes (95% CI −10.12 to −1.87; *p* < 0.010). We were unable to exclude increased harms from tourniquet use. Pooled analysis showed post-operative pain score was higher in tourniquet group by 12.88 on 100-point scale (95% CI −1.25–27.02; *p = *0.070). Risk differences for wound infection, deep venous thrombosis and re-operation were 0.06 (95% CI −0.00–0.12; *p* = 0.070), 0.05 (95% CI −0.02–0.11; *p* = 0.150) and 0.03 (95% CI -0.03–0.09; *p* = 0.340).

**Conclusion:**

Tourniquet use was associated with a reduced length of procedure. It is possible that tourniquets also increase incidence of important complications, but the data are too sparse to draw firm conclusions. Methodological weaknesses of the included RCTs prevent any solid conclusions being drawn for outcomes investigated. Further studies are required to address these limitations.

**Supplementary Information:**

The online version contains supplementary material available at 10.1007/s00590-021-02957-7.

## Introduction

Tourniquets are commonly used during lower limb fracture fixation surgery [1–4]. They are thought to reduce intra-operative bleeding, improve surgical field of view and reduce surgical time [5–7]. However, by compressing the local tissues a tourniquet can cause venous stasis and ischaemia [8–10] which may increase the risk of venous thromboembolism, neurovascular injury, fracture non-union and wound complications including infection [10–15]. A tourniquet may also cause pain both intra and post-operatively, which may require increasing the depth of anaesthesia and higher doses of analgesia, respectively [16]. High levels of post-operative pain may limit early rehabilitation and increase patients’ length of stay in hospital [17].

Other possible effects of a tourniquet include increased serum levels of lactate, carbon dioxide, free radicals and prostaglandins as a result of local tissue hypoxia [16, 18, 19]. Local tissue hypoxia may also increase the risk of wound healing issues, such as dehiscence, in already traumatised soft tissues.

A 2019 review (search date 2017, five studies; N = 364) [20] reported on a subset of these outcomes. We have updated this review, including all data on possible benefits and harms of tourniquet use in lower limb fracture fixation surgery.

In this this study, we compare patient centred, surgical and biochemical outcomes following lower limb fracture fixation surgery performed with or without a tourniquet.

## Methods

### Data sources and search strategy

We adhered to Preferred Reporting Items for Systematic Review and Meta-Analysis (PRISMA) guidelines [21] with a protocol registered in the International Prospective Register of Systematic Reviews (PROSPERO; CRD42020209310). We searched the following databases from their inception up to 2 October 2020: Medline, Embase, Web of Science, Literatura Latino Americana em Ciências da Saúde (LILACS), African Journals Online (AJOL), and Cochrane Central Register of Controlled Trials (CENTRAL) and Database of Systematic Reviews. We had an a priori set out of preferred patient centred, surgical and biochemical outcomes (Box 1). We did not exclude studies that did not report these outcomes of interest.

The search strategy is shown in Supplementary Table S1. The search results were independently assessed for inclusion by two authors (MFA, FD). Initial screening was by title and abstract. Further screening of selected full texts determined eligibility. Bibliographies of included articles and prior systematic reviews and meta-analyses were manually scanned to identify missed relevant articles.

### Eligibility criteria

We included all randomised controlled trials (RCTs) which examined people undergoing lower limb fracture fixation surgery (population) and compared using a tourniquet (intervention) to one of the following (comparators):

1. No tourniquet
2. Placebo (e.g. sham tourniquet)
3. Alternative measure to improve surgical field of view or reduce intra-operative blood loss (e.g. tranexamic acid, controlled hypotension)

RCTs in English or with an accessible translation were included. Conference abstracts and animal studies were excluded. Disagreements about study eligibility were discussed with the senior author (PW).

### Data extraction and quality assessment

RCT data were independently extracted by two authors (MFA, FD) using a standardised data form. Methodological quality was independently assessed by two authors (MFA, FD) using the Cochrane Risk of Bias Tool Version 2 [22], using the trial’s primary outcome, or outcome included in the article title, or first reported outcome in the text of the paper, in this order. Authors were contacted by email when clarification of their methods was required or where precise values of study results were not provided in the article text.

### Data synthesis and statistical analysis

Summary measures were abstracted from papers as mean differences (MD) for continuous outcomes and risk ratios (RR) for binary outcomes, along with 95% confidence intervals (CI). Risk differences (RD) were used to report outcomes related to complications when no events occur in either arm in one or more RCT. Data on outcomes under investigation were pooled, where possible, using the results of the most common endpoint examined between studies, where possible subgroup analyses by age (< 60 and ≥60 years) and fracture type at presentation (open/closed) were planned. An inverse-variance method meta-analysis was implemented for data pooling, using a random-effects model. Review Manager 5.3 (RevMan version 5.3. Copenhagen: The Nordic Cochrane Centre, The Cochrane Collaboration, 2014) was used for model fitting and data presentation. Statistical significance was assessed at the 5% level and heterogeneity quantified using Higgins* I*^2^ test, with values interpreted in accordance with the Cochrane Handbook [23]. Narrative discussion has been provided where statistical analysis was not possible. Data values extracted directly from studies are presented as mean and a standard deviation (SD).

## Results

### Study identification and selection

**Fig. 1:**
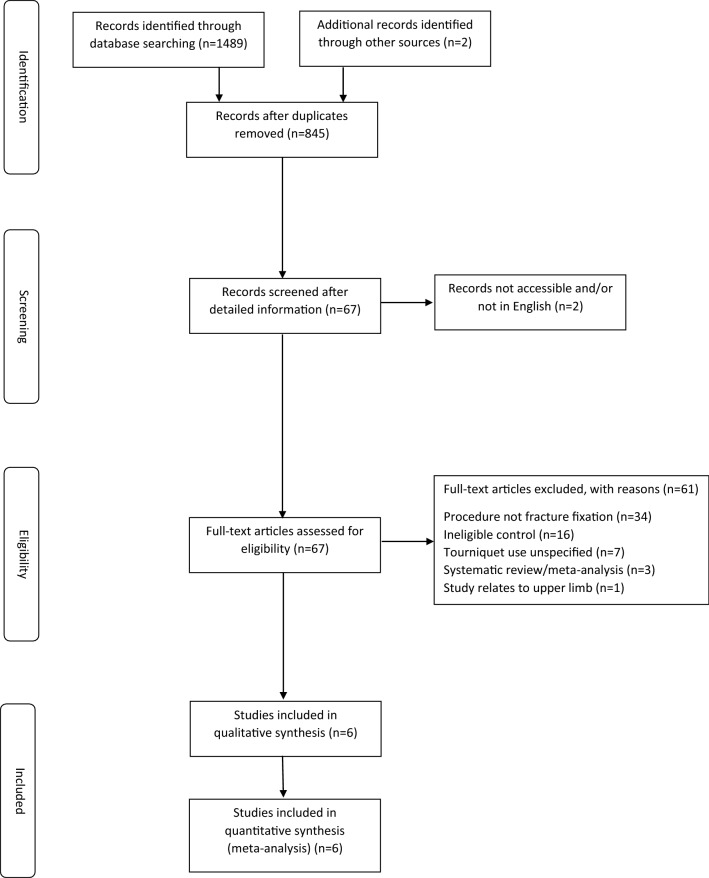
PRISMA flow diagram

We identified 845 potentially eligible studies. Following initial screening by title and abstract, 67 articles remained for full-text evaluation. One article potentially eligible for inclusion was unavailable in English [24]. Six articles met the inclusion criteria (Fig. 1 and Table 1) [25–30]. An excluded studies table is provided (Table S2).

**Table 1:** Description of included articles

Publication year	First author	Country	Study time period	Fracture type(s)	Total procedures (T/NT)^*^	Fracture Classification Breakdown (T/NT)^*^	Mean age (years) (range) (T/NT)^*^	Gender (male/female) (%) (T/NT)^*^	Outcome(s) investigated	Follow-up time points
1991	Salam	England	January 1987–January 1989	Closed tibial fractures	30/30	Not reported	32.2 (18 – 50) (combined)	T (90:10 N (87:13)	Pain, non-union, wound complications, blood loss, length of procedure, surgical perspective	Not reported
1993	Maffulli	Italy	January 1986–December 1990	Closed, isolated, displaced distal end of fibula fracture (AO† types 44-A1, 44-B1 or 44-C1)	40/40	T (24x 44-A1; 7x 44-B1; 9x 44-C1) NT (19x 44-A1; 5x 44-B1; 16x 44-C1)	T: 52 (18 – 60) NT: 50 (18 – 60)	T (83:17) NT (73:27)	Function, DVT §, wound complications, length of stay, length of procedure	Daily as inpatient, 6 weeks, 3 months, 6 months, 1 year, 6-monthly until metalwork removed. Mean duration of follow-up 18 months (range 9 – 32 months)
1997	Ömeroğlu	Turkey	Not reported	Malleolar fractures	16/16	T (3x type A; 7x type B; 6x type C) NT (1x type A; 7x type B; 8x type C)	T 40 (18 – 75) NT 37 (15 – 72)	T (63:37) NT (75:25)	Pain, length of procedure	24 and 48 hours post-operatively
2005	Konrad	Germany	December 2000–December 2001	Closed, displaced ankle fractures (AO 44-B, 44-C)	26/28	Not reported	T: 42.7 NT: 41.6	T (38:62) NT (39:61)	Pain, function, DVT§, wound complications, blood loss, length of stay, length of procedure, swelling	Day 2, day 5, week 6
2010	Saied	Iran	September 2007–May 2008	Acute extra-articular tibial fractures	65/73	Not reported	T: 39.7 (20 – 72) NT: 39.4 (20 – 72)	T (80:20) NT (79:21)	Pain, non-union, wound complications, blood loss, length of procedure, surgical perspective	24 hours and at 1 year
2019	Sim	England	August 2012–August 2015	Closed, isolated, displaced ankle fractures (Weber A, B or C)	94/94‡	T (80% Weber B; 20% Weber C) NT (66% Weber B; 34% Weber C)	T: 48.36 NT: 48.34	T (44:56) NT (37:63)	Blood loss, length of stay, length of procedure	Daily during inpatient and at 2 weeks

^*^T: tourniquet (patient group); NT: non-tourniquet (patient group)

^†^AO (arbeitsgemeinschaft für osteosynthesefragen)[43]

^‡^Intention to treat

^§^Deep vein thrombosis

**Box 1:** Patient centred, surgical and biochemical outcomes under investigation

*Patient outcomes*	
Pain	
Physical function	
Health-related quality of life	
Complications	
Cognitive function	
*Surgical Outcomes*	
Volume of blood loss	
Length of hospital stay	
Length of procedure	
Swelling	
Time to union	
Surgical field of view	
Volume of anaesthetic agent used	
*Biochemical Outcomes*	
Biochemical markers of inflammation and injury	
Electrolyte and acid–base balance	

### Study characteristics

Of the six RCTs included in the review, two were conducted in England [25, 30] and one each in Germany [28], Iran [29], Italy [26] and Turkey [27]. Four RCTs investigated ankle fractures [26–28, 30] and two RCTs investigated tibial fractures [25, 29]. There were data on 354 ankle fractures and 198 tibial fractures; 552 procedures in total. Three RCTs reported on mechanism of injury and 92% of the combined total number of fractures resulted from low-energy trauma [25, 26, 30]. Four RCTs included people with isolated injuries only [26, 27, 29, 30], one RCT excluded people who sustained an additional long bone fracture in the ipsilateral injured leg or any injury that would prevent partial weight bearing [28], and one RCT did not detail their inclusion criteria [25]. Four RCTs excluded people with open fractures [25, 26, 28, 30], two RCTs excluded people with diabetes mellitus [26, 28], and one RCT excluded people with peripheral neuropathy [27]. All participants underwent open reduction internal fixation using plates and screws. Participants in the control group underwent surgery without tourniquet in five RCTs [25–27, 29, 30] and using a sham tourniquet in one RCT [28]. Most participants were male (62%), and participant’s mean age ranged from 32 to 52 years. Number of participating surgeons varied between the RCTs, and all procedures were performed by one author in Salam [25], two authors in Maffulli [31] and by a team of surgeons in Ömeroğlu [27] and Saied [29]. Details of operating surgeons were not reported in the studies by Konrad [32] and Sim [30]. The RCTs reported on post-operative pain, blood loss, surgical perspective, length of procedure, length of stay, complications (wound, DVT, re-operation, non-union), swelling and function. None of the RCTs provided a sufficient level of detail to enable the subgroup analyses to be performed.

### Study quality

**Fig. 2:**
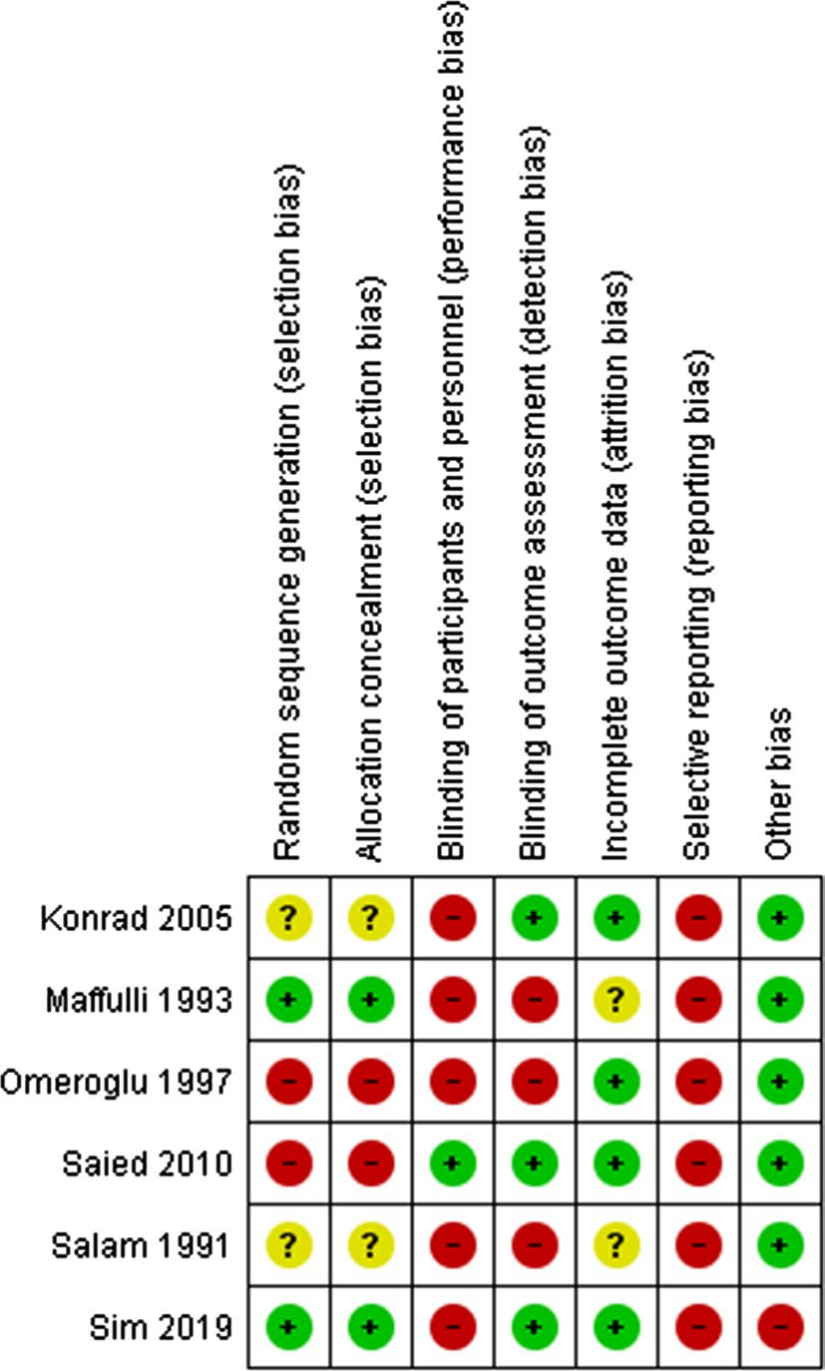
Risk of bias summary. 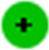 Low risk of bias, 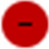 high risk of bias, and 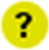 unclear risk of bias

The RCTs were of high risk of bias in multiple domains. Ömeroğlu [27] and Saied [29] had a high risk of selection bias due to patient allocation based on order of hospital admission and inadequate patient allocation concealment due to use of non-numbered, unsealed envelopes that were restored to their original condition once a cohort of 10 patients were allocated to a treatment, increasing the predictability of subsequent patient allocations. The authors of Saied [29] included the term double-blinded trial in their methods section but did not describe how this was performed. Detection bias was assessed based on length of procedure for the studies by Saied [29] and Maffulli [26] and outcomes blood loss, pain, swelling and length of stay in the studies by Salam [25], Ömeroğlu [27], Konrad [28], and Sim [30], respectively. Sim [30] performed adjusted analyses. None of the RCTs have published a publicly available trial protocol including those studies performed following the 2010 update of the Consolidated Standards of Reporting Trials (CONSORT) [33]. The RCT by Sim was judged to be at high risk for other sources of bias due to the differential crossover rate of 17% of patients to the tourniquet group. Figure 2 details the results of the bias assessment. Corresponding authors of three included studies were contacted to query on parts of their methods and results. No responses were received. One RCT [29] did not define the descriptive statistic used to represent data variability of the results however, this was interpreted to be one standard deviation.

### Protocol deviation

Our registered Prospero protocol (CRD42020209310) only specifies inclusion of RCTs in our review. We have included one study [27] where patient allocation to treatment was not strictly random and considered quasi-random [34]. This study allocated participants to intervention based on order of patient admission to the hospital. Odd numbered patients received surgery with a tourniquet, and even numbered patients received surgery without tourniquet. Inclusion of this study in our review does not affect its validity given that most of the included RCTs were also judged high risk of performance and detection bias.

### Patient outcomes

#### Post-operative pain

**Fig. 3:**
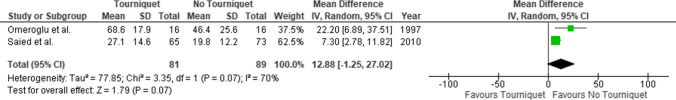
Mean difference and 95% CI for intensity of post-operative pain within 24 hours following surgery with versus without tourniquet

Four RCTs reported on post-operative pain however, only Ömeroğlu [27] and Saied [29] provided sufficient summary data to enable inclusion in a meta-analysis (*n* = 170). Ömeroğlu [27] measured pain using a 0–100 mm visual analogue scale (VAS), whereas Saied [29] utilised a 1–10 numerical rating scale. These values were converted and standardised to a 100-point scale format. Konrad [28] (*n* = 54) reported their results in graph format only. The authors reported that people who had surgery using a tourniquet had significantly greater pain, measured using a 0–10 numerical rating scale, at five days and six weeks post-operatively compared to the non-tourniquet group. Pain scores were approximated through direct measurement of the graph, and values at day two, day five and week six post-operatively were 3.4 versus 2.7, 2.1 versus 1.4, and 1.2 versus 0.4, respectively (tourniquet versus non-tourniquet). Salam [25] (*n* = 60) reported that plaster casts required removal due to pain in 6/30 (20%) of participants in the tourniquet group compared to none in the non-tourniquet group. A post hoc Fisher's exact test comparing these two groups’ results for illustrative purposes revealed *p* < 0.050. Ömeroğlu assessed pain using two outcome measures, 0–100 mm VAS and present pain intensity scale [35], at four different times; 24 and 48 hours post-operatively one hour before and after patients received analgesia. Differences in present pain intensity scale scores between patient groups were statistically significant at all time points except at 24 hours post-operatively after analgesia. However, differences in VAS results between patient groups were statistically significant at all time points. The VAS measurement reported by patients one hour before receiving analgesia in the RCT by Ömeroğlu was used for the meta-analysis due to potential differences in analgesic consumption between patient groups. No statistically significant differences were observed between patient groups (figure 3). There was substantial between-study heterogeneity (*I*^2^ = 70%) however, this was not statistically significant (*p* = 0.070).

### Function

Two RCTs [26, 28] reported on range of motion (ROM) however, this was assessed at substantially different time points and therefore it was not appropriate to pool these [26, 28]. Konrad [28] (*n* = 56), in a RCT of ankle fractures, found no differences in ankle ROM at day five and week six post-operatively between tourniquet and non-tourniquet patient groups (33° ± 10° versus 36° ± 10°; *p* = 0.25, and 49° ± 10° versus 56° ± 15°; *p* = 0.06, respectively). Maffulli [26] (*n* = 80) reported the results for ankle range of motion at patients’ latest follow-up evaluation which took place at a combined average of 18 months post-operatively (range 9–32 months). Similarly, no differences in ankle ROM were found between tourniquet and non-tourniquet patient groups; 63° ± 12° (range 50°–81°) and 67° ± 14° (range 55°–80°), respectively. Maffulli also compared time to return to full-time employment and full weight bearing between patient groups. Although there were no differences observed in time to full weight bearing between patient groups (57±10 and 51±10 days; tourniquet versus no tourniquet), people who had surgery without a tourniquet returned to work earlier than people in the group who underwent surgery with a tourniquet; 55±9 days (range 45–63) versus 62±13 days (range 42–74), *p* < 0.050.

### Non-union

**Fig. 4:**
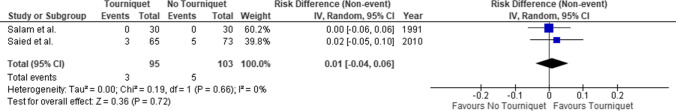
Risk difference and 95% CI of non-union following fracture fixation surgery with versus without tourniquet

Two RCTs [25, 29] compared non-union between patient groups. Saied [29] (*n* = 138) defined occurrence of union when the patient was able to walk without pain along with radiographic evidence of continuity of the cortex with lack of a visible fracture line. Mean time to union was similar between tourniquet and non-tourniquet groups (4.45 ± 1.22 versus 4.79 ± 1.46 months; *p* = 0.223). Prevalence of non-union between participants following surgery with and without tourniquet at one year was very similar − 3/65 (4.6%) versus 5/73 (6.8%) people, respectively. Salam [25] (*n* = 60) reported that fractures of all participants united. No differences were observed between patient groups (RD 0.01; 95% CI −0.04–0.06; *p* = 0.720) (figure 4), and there were no significant differences in heterogeneity between the included RCTs (*I*^2^ = 0%; *p* = 0.660).

### Deep vein thrombosis

**Fig. 5:**
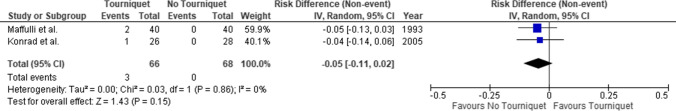
Risk difference and 95% CI of deep vein thrombosis following fracture fixation surgery with versus without tourniquet

The RCTs by Konrad [28] and Maffulli [26] reported on DVT (*n* = 134). None of the 68 patients who had surgery without a tourniquet suffered a DVT however, this occurred in three of the 66 patients who had surgery with a tourniquet. These were confirmed by venography and Doppler scans in the RCT by Maffulli however, the diagnostic method in the RCT by Konrad is not reported. No differences were found between patient groups (RD −0.05, 95% CI − 0.11–0.02; *p* = 0.150) (Fig. 5). There was no significant evidence of heterogeneity between included RCTs (*I*^2^ = 0%; *p* = 0.860).

### Wound complications

**Fig. 6:**
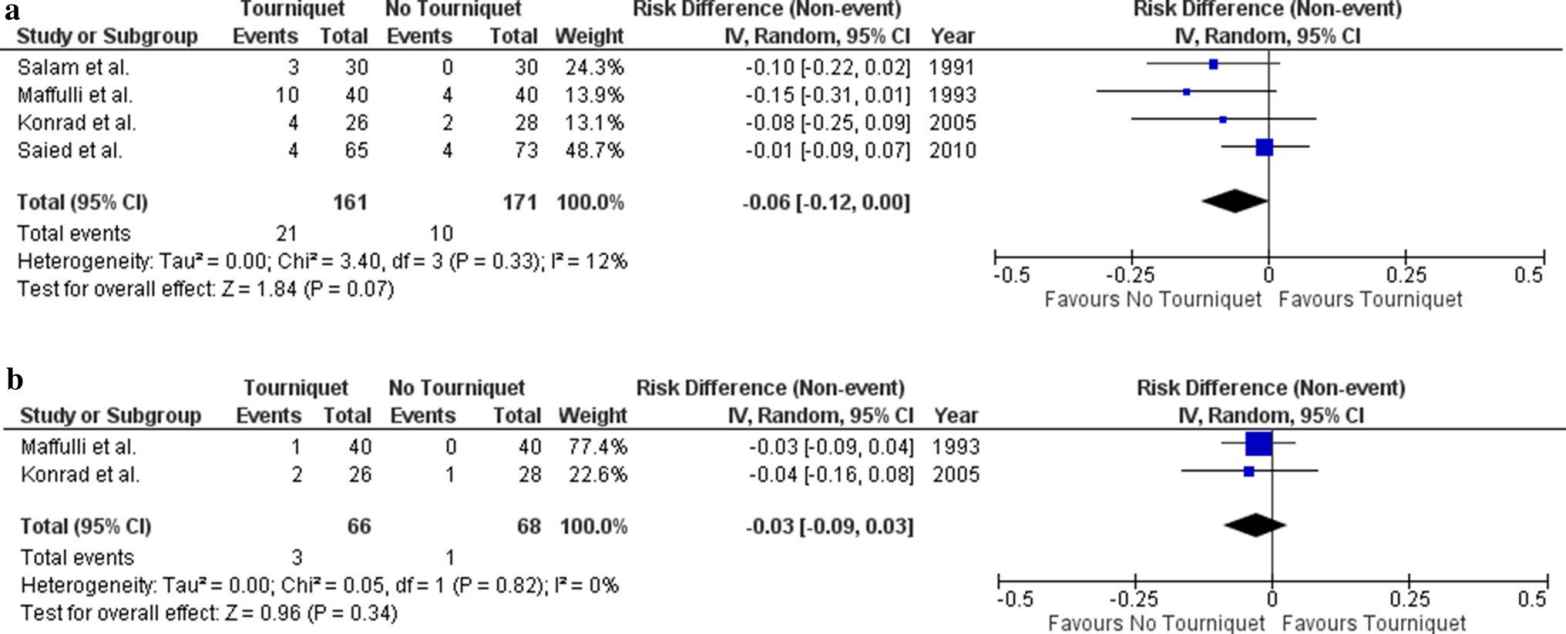
**a** Risk difference and 95% CI of wound infection following fracture fixation surgery with versus without tourniquet. **b** Risk difference and 95% CI of re-operation following fracture fixation surgery with versus without tourniquet

There were four RCTs [25, 26, 28, 29] which reported on wound infection (*n* = 332) with varying definitions used between studies. For the purposes of the meta-analysis, wound complications managed with antibiotics were considered infection related. Wound infections occurred in 21 of 161 patients (13%) who had surgery with a tourniquet and in 10/171 (6%) participants who had surgery without a tourniquet. The risk difference for wound infection between people who underwent fracture fixation surgery with versus without tourniquet is −0.06 (95% CI −0.12–0.00; *p* = 0.070) (Fig. 6a). There was insignificant heterogeneity between the pooled RCTs (*I*^2^ = 12%; *p* = 0.330). The RCT by Salam [25] reported on non-infected wound complications and found a higher incidence in the tourniquet patient group (3/30 versus 0/30 participants).

Re-operation was reported in two RCTs (*n* = 134) [26, 28]. Wound infection was the indication for all procedures. These are a subset of all reported infection cases. The risk difference for re-operation following fracture fixation surgery with versus without tourniquet is −0.03 (95% CI −0.09–0.03; *p* = 0.340) (Fig. 6b). No significant evidence of heterogeneity between the RCTs was observed (*I*^2^ = 0%; *p* = 0.820).

### Surgical outcomes

#### Blood loss

Four of the included RCTs reported on blood loss however, only one RCT quantified volumes [25, 28–30]. Saied [29] (*n* = 138) compared volumes of surgical drain contents in the first 24 hours following surgery between the two patient groups. Patients in the non-tourniquet group had a reduced content volume in their surgical drains compared to patients in the tourniquet group (21.20 ± 7.44 ml versus 23.47 ± 6.44 ml; *p* = 0.03). Salam [25] (*n* = 60) reported that the two patient groups did not differ in blood loss. No supportive data are presented. Konrad [28] (*n* = 54) reported that none of the patients in their trial had a significant decrease in haemoglobin value two days following surgery and none required a blood transfusion post-operatively. Sim [30] (*n* = 188) reported on complications and included one event in the non-tourniquet group where a person lost 250 ml of blood and in another unrelated event, a patient sustained a vascular injury intra-operatively.

### Length of stay

**Fig. 7:**
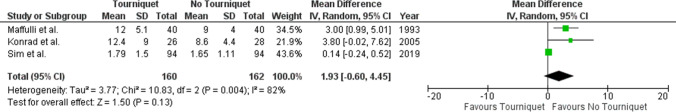
Mean difference and 95% CI in length of stay between patients receiving surgery with versus without tourniquet

Three RCTs compared length of stay between patient groups (*n* = 322) [26, 28, 30]. For the meta-analysis, length of stay including delays related to complications was used as this is reflective of real-life practice. We used the results of the intention to treat analysis from Sim [30] for the meta-analysis. Pooled analysis demonstrated that people had a longer length of hospital stay after fracture fixation if their surgery was performed with, instead of without, a tourniquet however, this result was not statistically (MD 1.93 days, 95% CI −0.60–4.45; *p* = 0.130) (Fig. 7). There was considerable statistically significant heterogeneity between the included RCTs (*I*^2^ = 82%; *p* < 0.010).

### Length of procedure

**Fig. 8:**
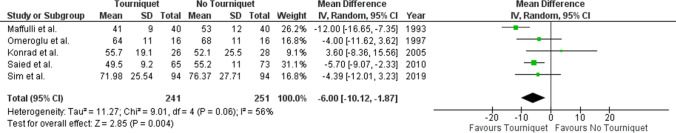
Mean difference and 95% CI for length of procedure when performed with versus without tourniquet

All included RCTs reported on outcome length of procedure (*n* = 492). Salam [25] state that the two groups did not differ with respect to operation time. No supportive data are presented. Figure 8 reports the mean difference (95% CI) in the length of procedure comparing lower limb fracture fixation performed with and without tourniquet. Pooled analyses of five RCTs demonstrated a reduction in duration of procedure with the use of tourniquet (MD –6 minutes, 95% CI −10.12 to −1.87; *p* < 0.010). There was substantial heterogeneity between included RCTs however, this was not statistically significant (*I*^2^ = 56%; *p* = 0.060)

### Swelling

Only the RCT by Konrad [28] investigated this outcome (*n* = 54). They measured the circumference of the patient’s injured and uninjured ankle at the level of the malleoli and compared the difference in these values between the tourniquet and non-tourniquet groups at days two and five, and week six post-operatively. No differences in ankle circumference were found two days post-operatively between the tourniquet and non-tourniquet patient groups (23 mm and 26 mm, respectively; *p* = 0.73). However, by the fifth post-operative day, average ankle circumference increased to 26 mm in the tourniquet group and decreased to 19 mm in the non-tourniquet group (*p* < 0.01). It is reported that the difference in values between the two patient groups remained constant and significantly different at 6 weeks post-operatively (*p* < 0.01) however, these data were presented in graph format only. Estimates of these values were obtained by direct measurement of the graph and ankle circumference was approximately 19 mm and 10 mm in the tourniquet and non-tourniquet patient groups, respectively. These differences remained statistically significant in a separate analysis that excluded patients who suffered from wound infections and deep vein thrombosis (DVT) which may have contributed to swelling.

### Surgical perspective

No RCTs reported on surgical field of view. Two RCTs, however, did consider the surgical perspective. Salam [25] (*n* = 60) state that the two groups did not differ with respect to technical difficulties. No supportive data are presented. Saied [29] (*n* = 138) surveyed surgeons who performed fracture fixation without tourniquet and established that most surgeons (91.8%) would avoid using a tourniquet for a similar procedure in future.

### Other outcomes

No RCTs reported on health-related quality of life, cognitive function, volume of anaesthetic agent used, biochemical markers of inflammation and injury, and electrolyte and acid–base balance.

## Discussion

This is the most comprehensive meta-analysis comparing the effects of lower limb fracture surgery performed with versus without tourniquet. However, although we abstracted data from RCTs, the methodological quality of most of the trials included in our review was low and this must be considered when interpreting the results of our study. All RCTs were judged to be high risk of bias in at least two domains (Fig. 2) and most of the RCTs did not blind patients and surgeons, and not all outcomes under investigation were assessed by blinded assessors. Furthermore, all the RCTs contained small numbers of patients.

Our review found no statistically significant difference in post-operative pain at 24 hours between patient groups (MD 12.88 mm, 95% CI −1.25–27.02; *p* = 0.070), although a clinically important difference (previously defined as 12 mm for acute pain) cannot be ruled out [36]. The two groups did not differ with respect to range of motion and time to full weight bearing. However, patients who underwent surgery without a tourniquet returned to full-time employment relatively quicker (55±9 days versus 62±13 days, *p* < 0.05). Pooled analysis did not show any statistically significant differences in wound complications (RD 0.06, 95% CI −0.00–0.12; *p* = 0.070), DVT (RD 0.05, 95% CI −0.02–0.11; *p* = 0.150) and re-operation (RD 0.03, 95% CI −0.03–0.09; *p* = 0.340) between the patient groups. Nevertheless, the point estimate for risk difference in each case is likely to be clinically important. Much more data would be needed to demonstrate if such differences in these categorical variables were truly present. There were also no differences in prevalence of non-union. Blood loss and length of hospital stay did not differ between groups. Pooled analysis for length of procedure favoured tourniquet use (MD 6 minutes, 95% CI −10.12 to −1.87; *p* = 0.004). Tourniquet use was associated with greater ankle swelling up to six weeks post-operatively (10 mm versus 19 mm, *p* < 0.01) compared to surgery without a tourniquet. There were no differences in the results for surgical perspective between procedures performed with and without a tourniquet.

Patients operated on without a tourniquet experienced relatively less post-operative pain at 24 hours (MD 12.88 mm). Although this result was not statistically significant (*p* = 0.070), the confidence interval for the overall effect estimate is wide (95% CI −1.25–27.02) limiting the precision of our results. This is typically generated by small sample sizes and high dispersion such as pooling the results of tibial and ankle fractures together. It is possible that the true MD value may exceed the minimum clinically important difference for VAS which is approximately 10–20 mm [36–38]. The shorter procedure duration observed when a tourniquet was used may be due to an improved surgical field of view however, we could not identify any RCTs which robustly assessed this outcome in lower limb fracture fixation surgery. Furthermore, none of the included RCTs apart from maybe one-blinded surgeons to tourniquet status introducing possibility of performance bias. Tourniquet use may place pressure on surgeons to complete procedures relatively more quickly given that tourniquet application time should not exceed 1.5–2 hours [39]. In other settings where tourniquets are used such as in knee replacement surgery, and knee and ankle arthroscopy, studies have reported no differences in surgical field of view between patients undergoing surgery with or without tourniquet [40–42]. Furthermore, tourniquets are believed to improve surgical field of view by reducing intra-operative bleeding however, the included RCTs in our study did not find differences in blood loss between patient groups. It is also possible that any reduction in intra-operative bleeding may have been offset by greater post-operative blood loss with the use of a tourniquet [15]. We found a mean difference of six minutes in length of procedure (95% CI 1.87 – 10.12) however, this may not have taken into account any additional time in theatre required for patient preparation and set-up, which was shown to be longer with the use of tourniquet in one RCT (13 ± 6.3 versus 8 ± 6.8 minutes; *p* = 0.03) [26].

There are additional weaknesses of the included RCTs to those already described. The definitions of non-union and wound infection varied between the trials and were not consistent with internationally accepted criteria. Also, some of the included RCTs applied restrictive and arbitrary inclusion criteria such as non-diabetic patients only and smokers of less than 5 cigarettes per day which reduces the generalisability of their results. To enhance pragmatism, we pooled the results of RCTs investigating tibial and ankle fractures and analysed these in combination. However, this pooling contributed to the heterogeneity observed in our meta-analysis. Furthermore, it is possible that findings may vary between tibial and ankle fractures.

We could not identify any RCT data for several of the outcomes under investigation in this review including cognitive function, health-related quality of life, volume of anaesthetic agent used, biochemical markers of inflammation and injury, and electrolyte and acid–base balance. There were also very limited evidence relating to surgical field of view and blood loss, and no data on patient reported outcome measures of function or post-operative pain at longer-term endpoints. Furthermore, despite pooling studies investigating various fractures of the lower limb, complications are rare events, and our meta-analysis is underpowered to detect any potential difference for these outcomes. For example, only three of 134 patients developed a deep vein thrombosis in the pooled results and much larger studies would be required to determine whether tourniquet uses affects the incidence of adverse events. These several major limitations of the evidence base should be urgently addressed in a large, multicentre RCT. This would also enable any results to be pragmatic and reflective of practice between various units. However, complications are rare events (approximately 1–2%) and a trial of > 1000 patients would be required to detect differences between groups.

A 2019 review [20] that included five of the six studies in this review found similar results for complications and length of procedure. In contrast to our review, the authors concluded that tourniquets increased post-operative pain at 24 hours and length of hospital stay. Despite pooling results of studies investigating different fractures of the lower limb, the authors employed a fixed-effects meta-analysis model which resulted in their finding for post-operative pain being statistically significant. The previous review primarily investigated post-operative pain and post-operative complications, whereas our review included a range of additional outcomes. We have also pooled the results for most outcomes under investigation where data were available including length of stay and length of procedure. Furthermore, we analysed outcomes of procedure related complications individually rather than collectively to evaluate effect estimates for each possible complication independently. There are also some differences in data extraction from the included studies in the two reviews. More specifically, the total number of patients within each group in their pooled analysis for outcome post-operative pain is incorrect.

## Limitations

The main limitations of this review are attributed to the quality and quantity of the included RCTs. These were all deemed high risk of bias in multiple domains mainly performance and reporting biases. Also, all included RCTs were carried out at a single-centre and procedures were performed by a selected group of surgeons limiting the generalisability of our results. We attempted to address this by pooling all the data together including RCTs investigating ankle and tibial fractures. The consequential heterogeneity of the data, however, does reduce the strength of our conclusions.

## Conclusions

In summary, a tourniquet reduces the duration of surgery however, we did not find evidence for a statistically significant difference in post-operative pain at 24 hours, blood loss, function, length of stay, and complications between patient groups, although a clinically important difference cannot be ruled out based on the current evidence. Numbers of complications were low due to relatively small sample sizes. At this time, it is not possible to draw firm conclusions on the use of a tourniquet in lower limb fracture fixation surgery. However, surgery without a tourniquet helps avoid any potential harms associated with tourniquet use and is equally feasible. In light of our study results, patients should be made aware of the potential benefits and harms of using a tourniquet for their fracture fixation surgery.

## Future directions

This review has highlighted a paucity of high-quality RCTs investigating outcomes following lower limb fracture surgery performed using a tourniquet. Further research on this subject is urgently needed given the limitations of the existing evidence and a large, pragmatic RCT involving all levels of surgeons and hospitals across the country should be performed. Multicentre involvement would help to ensure a sufficient sample size, enabling meaningful results, particularly for complication outcomes. This proposed study should also address the identified issues which contributed to a high risk of bias in the existing RCTs. The results of this study would establish the comparative benefits and risks of tourniquet use.

## Supplementary Information

Below is the link to the electronic supplementary material.

Supplementary file1 (DOCX 13 kb)

Supplementary file2 (DOCX 29 kb)

## References

[1] Cunningham L, McCarthy T, O'Byrne J (2013). A survey of upper and lower limb tourniquet use among Irish orthopaedic surgeons. Ir J Med Sci.

[2] Boya H, Tuncali B, Ozcan O, Arac S, Tuncay C (2016). Practice of tourniquet use in Turkey: a pilot study. Acta Orthop Traumatol Turc.

[3] Younger AS, Kalla TP, McEwen JA, Inkpen K (2005). Survey of tourniquet use in orthopaedic foot and ankle surgery. Foot Ankle Int.

[4] Kalla TP, Younger A, McEwen JA, Inkpen K (2003). Survey of tourniquet use in podiatric surgery. J Foot Ankle Surg.

[5] Sato J, Ishii Y, Noguchi H, Takeda M (2012). Safety and efficacy of a new tourniquet system. BMC Surg.

[6] Ishii Y, Noguchi H, Takeda M (2010). Clinical use of a new tourniquet system for foot and ankle surgery. Int Orthop.

[7] Estebe JP, Davies JM, Richebe P (2011). The pneumatic tourniquet: mechanical, ischaemia-reperfusion and systemic effects. Eur J Anaesthesiol.

[8] Yassin MDMMI, Harkin MDDW, Barros D’Sa MDAAB, Halliday PDMI, Rowlands MDBJ (2002). Lower limb ischemia-reperfusion injury triggers a systemic inflammatory response and multiple organ dysfunction. World J Surg.

[9] Clarke MT, Longstaff L, Edwards D, Rushton N (2001). Tourniquet-induced wound hypoxia after total knee replacement. J Bone Joint Surg Br.

[10] McMillan TE, Gardner T, Johnstone AJ (2020). Current concepts in tourniquet uses. Surgery (Oxford).

[11] Deloughry JL, Griffiths R (2009). Arterial tourniquets. Contin Edu Anaesth Crit Care Pain.

[12] Van der Spuy L (2012). Complications of the arterial tourniquet. South Afr J Anaesth Analg.

[13] Wong S, Irwin MG (2015). Procedures under tourniquet. Anaesth Intensive Care Med.

[14] Huwae TECJ, Ratnawati R, Sujuti H, Putra BSS, Putera MA, Hidayat M (2020). The effect of using torniquets on fracture healing disorders: a study in wistar strain rats (Rattus norvegicus). Int J Surg Open.

[15] Zhang W, Li N, Chen S, Tan Y, Al-Aidaros M, Chen L (2014). The effects of a tourniquet used in total knee arthroplasty: a meta-analysis. J Orthop Surg Res.

[16] Kumar K, Railton C, Tawfic Q (2016). Tourniquet application during anesthesia: "What we need to know?". J Anaesthesiol Clin Pharmacol.

[17] Morrison RS, Magaziner J, McLaughlin MA, Orosz G, Silberzweig SB, Koval KJ (2003). The impact of post-operative pain on outcomes following hip fracture. Pain.

[18] Cheng YJ, Chien CT, Chen CF (2003). Oxidative stress in bilateral total knee replacement, under ischaemic tourniquet. J Bone Joint Surg Br.

[19] Girardis M, Milesi S, Donato S, Raffaelli M, Spasiano A, Antonutto G (2000). The hemodynamic and metabolic effects of tourniquet application during knee surgery. Anesth Analg.

[20] Praestegaard M, Beisvag E, Erichsen JL, Brix M, Viberg B (2019). Tourniquet use in lower limb fracture surgery: a systematic review and meta-analysis. Eur J Orthop Surg Traumatol Orthop Traumatol.

[21] Moher D, Liberati A, Tetzlaff J, Altman DG, Group P (2009). Preferred reporting items for systematic reviews and meta-analyses: the PRISMA statement. PLoS Med.

[22] Higgins JPT, Altman DG, Gøtzsche PC, Jüni P, Moher D, Oxman AD (2011). The cochrane collaboration’s tool for assessing risk of bias in randomised trials. BMJ.

[23] Higgins J, Altman D, Higgins J, Green S (2008). Assessing risk of bias in included studies. Cochrane Handbook for Systematic Reviews of Interventions 510.

[24] Xie CY, Xiao JF, Zhao ZL (2015). Protective effect of ulinastatin against activation of tourniquet-induced platelet mitochondria apoptotic signaling. Zhongguo Shi Yan Xue Ye Xue Za Zhi.

[25] Salam AA, Eyres KS, Cleary J, el Sayed HH (1991). The use of a tourniquet when plating tibial fractures. J Bone Joint Surg Br.

[26] Maffulli N, Testa V, Capasso G (1993). Use of a tourniquet in the internal fixation of fractures of the distal part of the fibula. A prospective, randomized trial. J Bone Joint Surg Am.

[27] Omeroglu H, Gunel U, Bicimoglu A, Tabak AY, Ucaner A, Guney O (1997). The relationship between the use of tourniquet and the intensity of postoperative pain in surgically treated malleolar fractures. Foot Ankle Int.

[28] Konrad G, Markmiller M, Lenich A, Mayr E, Ruter A (2005). Tourniquets may increase postoperative swelling and pain after internal fixation of ankle fractures. Clin Orthop Relat Res.

[29] Saied A, Zyaei A (2010). Tourniquet use during plating of acute extra-articular tibial fractures: effects on final results of the operation. J Trauma.

[30] Sim J, Grocott N, Majeed H, McClelland D (2019). Effect on hospital length of stay of tourniquet use during internal fixation of ankle fractures: randomized controlled trial. J Foot Ankle Surg.

[31] Maffulli N, Testa V (1993). Use of a tourniquet in the internal fixation of fractures of the distal part of the fibula. A prospective, randomized trial. J Bone Joint Surg Ser A.

[32] Konrad G, Markmiller M, Lenich A, Mayr E, Rüter A (2005). Tourniquets may increase postoperative swelling and pain after internal fixation of ankle fractures. Clin Orthop Relat Res.

[33] Moher D, Hopewell S, Schulz KF, Montori V, Gøtzsche PC, Devereaux PJ (2010). CONSORT 2010 explanation and elaboration: updated guidelines for reporting parallel group randomised trials. BMJ.

[34] Lefebvre C, Manheimer E, Glanville J, Higgins J, Green S (2011). Chapter 6: Searching for studies. Cochrane Handbook for Systematic Reviews of Interventions 510.

[35] Melzack R (1987). The short-form McGill pain questionnaire. Pain.

[36] Kelly AM (2001). The minimum clinically significant difference in visual analogue scale pain score does not differ with severity of pain. Emerg Med J.

[37] Jensen MP, Chen C, Brugger AM (2002). Postsurgical pain outcome assessment. Pain.

[38] Bird SB, Dickson EW (2001). Clinically significant changes in pain along the visual analog scale. Ann Emerg Med.

[39] Deloughry JL, Griffiths R (2009). Arterial tourniquets. Contin Edu Anaesth Crit Care Pain.

[40] Zaidi R, Hasan K, Sharma A, Cullen N, Singh D, Goldberg A (2014). Ankle arthroscopy: a study of tourniquet versus no tourniquet. Foot Ankle Int.

[41] Ejaz A, Laursen AC, Kappel A, Laursen MB, Jakobsen T, Rasmussen S (2014). Faster recovery without the use of a tourniquet in total knee arthroplasty. Acta Orthop.

[42] Reda W, ElGuindy AMF, Zahry G, Faggal MS, Karim MA (2016). Anterior cruciate ligament reconstruction; is a tourniquet necessary? a randomized controlled trial. Knee Surg Sports Traumatol Arthrosc.

[43] Müller ME, Nazarian S, Koch P, Schatzker J. (2012) The comprehensive classification of fractures of long bones. Springer Science & Business Media

