# Supplementary material for: The risks associated with tourniquet use in lower limb trauma surgery: a systematic review and meta-analysis
Source: Eur J Orthop Surg Traumatol. 2021 Apr 1;31(5):967–79. doi: 10.1007/s00590-021-02957-7 (PMC8233247; doi:10.1007/s00590-021-02957-7)
Supplement: Supplementary file 1 — Supplementary file1 (DOCX 13 kb) [file 590_2021_2957_MOESM1_ESM.docx]

| Medline and Embase | (fracture fixation/ or fracture*.ti,ab,kw. or Lower Extremity/ or femoral Fractures/ or Tibial Fractures/ or Patella/ or Ankle Fractures/ or Fibula/ or Foot Bones/ or Talus/ or fixation.ti,ab,kw. or plat*.ti,ab,kw. or calcan*.ti,ab,kw. or nail.ti,ab,kw. or ORIF.ti,ab,kw) **AND** (tourniquets/ or Esmarch.ti,ab,kw. or Lofquist.ti,ab,kw. or cuff.ti,ab,kw.) **AND** (randomi?e*.ti,ab,kw. or trial.ti,ab,kw. or randomi?ed controlled trial.pt.) |
| --- | --- |
| The Cochrane Library | (MeSH descriptor: [Fracture Fixation] explode all trees  OR  (fracture*):ti,ab,kw  OR MeSH descriptor: [Lower Extremity] explode all trees  OR MeSH descriptor: [Femoral Fractures] explode all trees OR  MeSH descriptor: [Tibial Fractures] explode all trees OR MeSH descriptor: [Patella] explode all trees OR MeSH descriptor: [Ankle Fractures] explode all trees OR MeSH descriptor: [Fibula] explode all trees OR MeSH descriptor: [Foot Bones] explode all trees OR MeSH descriptor: [Talus] explode all trees OR (plat*):ti,ab,kw OR (calcan*):ti,ab,kw OR (fixation):ti,ab,kw OR (nail):ti,ab,kw OR (ORIF):ti,ab,kw)  **AND**  (MeSH descriptor: [Tourniquets] explode all trees OR (Esmarch):ti,ab,kw OR (Lofquist):ti,ab,kw OR (cuff):ti,ab,kw)  **AND** ((randomi?e*):ti,ab,kw OR (trial):ti,ab,kw OR ("randomi?ed controlled trial"):pt) |
| Web of Science | TS=(fracture* near/5 (femur or femoral or tibia* or patella* or ankle or fibula* or tal* or calcan* or foot or feet))  **OR**  TS=(fixation)  **OR**  TS=(nail*)  **OR**  TS=(ORIF)  **AND**  TS=(tourniquets or esmarch or lofquist or cuff)  **AND**  TS=(randomi?e* or trial) |
| Literatura Latino Americana em Ciências da Saúde (LILACS) | (MH:"Fracture Fixation" OR fracture$ OR MH:"Lower Extremity" OR MH:"Femoral Fractures" OR MH:"Tibial Fractures" OR MH:"Patella" OR MH:"Ankle Fractures" OR MH:"Talus" OR MH:"Fibula" OR MH:"Foot bones" OR "fixation" OR plat$ OR calcan$ OR nail$ OR "ORIF") **AND** (MH:"Tourniquets" OR "esmarch" OR "lofquist" OR "cuff") **AND** (random$ OR "trial") |
| African Journals Online (AJOL) | tourniquet fracture |

Supplementary Table 1: Medical subject headings and free words used in the literature search across the six databases.
