# Supplementary material for: The risks associated with tourniquet use in lower limb trauma surgery: a systematic review and meta-analysis
Source: Eur J Orthop Surg Traumatol. 2021 Apr 1;31(5):967–79. doi: 10.1007/s00590-021-02957-7 (PMC8233247; doi:10.1007/s00590-021-02957-7)
Supplement: Supplementary file 2 — Supplementary file2 (DOCX 29 kb) [file 590_2021_2957_MOESM2_ESM.docx]

| **Procedure not fracture fixation** |
| --- |
| 1. Stetzelberger V, Obertacke U, Jawhar A, V. S, U. O. Tourniquet application during TKA did not affect the accuracy of implant positioning: a randomized clinical trial. Knee Surg Sports Traumatol Arthrosc. 2018;26(6):1728–36. 2. Husted H, Toftgaard Jensen T, H. H. Influence of the pneumatic tourniquet on patella tracking in total knee arthroplasty: a prospective randomized study in 100 patients. J Arthroplasty. 2005;20(6):694–7. 3. Tarwala R, Dorr LD, Gilbert PK, Wan Z, Long WT. Tourniquet use during cementation only during total knee arthroplasty: a randomized trial. Clin Orthop Relat Res. 2014;472(1):169-74. 4. Ledin H, Aspenberg P, Good L, H. L, P. A. Tourniquet use in total knee replacement does not improve fixation, but appears to reduce final range of motion. Acta Orthop. 2012 Oct;83(5):499–503. 5. Chiu FY, Hung SH, Chuang TY, Chiang SC. The impact of exsanguination by Esmarch bandage on venous hemodynamic changes in total knee arthroplasty - A prospective randomized study of 38 knees. knee. 2012;19(3):213‐217. 6. Ejaz A, Laursen AC, Jakobsen T, Rasmussen S, Nielsen PT, Laursen MB. Absence of a Tourniquet Does Not Affect Fixation of Cemented TKA: A Randomized RSA Study of 70 Patients. J Arthroplasty. 2015;30(12):2128-32. 7. Molt M, Harsten A, Toksvig-Larsen S. The effect of tourniquet use on fixation quality in cemented total knee arthroplasty a prospective randomized clinical controlled RSA trial. Knee. 2014;21(2):396-401. 8. Tarwala R, Dorr LD, Gilbert PK, Wan Z, Long WT. Tourniquet use during cementation only during total knee arthroplasty: a randomized trial. Clin Orthop Relat Res. 2014;472(1):169-74. 9. Dennis DA, Kittelson AJ, Yang CC, Miner TM, Kim RH, Stevens-Lapsley JE. Does Tourniquet Use in TKA Affect Recovery of Lower Extremity Strength and Function? A Randomized Trial. Clin Orthop Relat Res. 2016;474(1):69-77. 10. Huang Z, Xie X, Li L, Huang Q, Ma J, Shen B, et al. Intravenous and Topical Tranexamic Acid Alone Are Superior to Tourniquet Use for Primary Total Knee Arthroplasty: A Prospective, Randomized Controlled Trial. J Bone Joint Surg Am. 2017;99(24):2053-61. 11. Worland RL, Arredondo J, Angles F, Lopez-Jimenez F, Jessup DE. Thigh pain following tourniquet application in simultaneous bilateral total knee replacement arthroplasty. J Arthroplasty. 1997;12(8 CC-Anaesthesia CC-Bone, Joint and Muscle Trauma CC-Pain, Palliative and Supportive Care):848‐852. 12. Hakkalamani S, Clark V, Pradhan N. Short versus standard duration tourniquet use during Total Knee Replacement A pilot study. Acta Orthop Belg. 2015 Mar;81(1):52–6. 13. Jawhar A, Stetzelberger V, Kollowa K, Obertacke U. Tourniquet application does not affect the periprosthetic bone cement penetration in total knee arthroplasty. KNEE Surg Sport Traumatol Arthrosc. 2019 Jul;27(7):2071–81. 14. Ejaz A, Laursen AC, Kappel A, Laursen MB, Jakobsen T, Rasmussen S, et al. Faster recovery without the use of a tourniquet in total knee arthroplasty. ACTA Orthop. 2014 Aug;85(4):422–6. 15. Zhang W, Li N, Chen S, Tan Y, Al-Aidaros M, Chen L. The effects of a tourniquet used in total knee arthroplasty: a meta-analysis. J Orthop Surg Res. 2014 Mar;9. 16. Vertullo CJ, Nagarajan M. Is cement penetration in TKR reduced by not using a tourniquet during cementation? A single blinded, randomized trial. J Orthop Surg. 2017 Jan;25(1). 17. Zhang W, Liu A, Hu D, Tan Y, Al-Aidaros M, Pan Z. Effects of the timing of tourniquet release in cemented total knee arthroplasty: a systematic review and meta-analysis of randomized controlled trials. J Orthop Surg Res. 2014 Dec;9. 18. Huang Z, Ma J, Zhu Y, Pei F, Yang J, Zhou Z, et al. Timing of Tourniquet Release in Total Knee Arthroplasty. Orthopedics. 2015 Jul;38(7):445–51. 19. Vaishya R, Agarwal AK, Vijay V, Tiwari MK. Short term outcomes of long duration versus short duration tourniquet in primary total knee arthroplasty: A randomized controlled trial. J Clin Orthop Trauma. 2018;9(1):46-50. 20. Mittal R, Ko V, Adie S, Naylor J, Dave J, Dave C, et al. Tourniquet application only during cement fixation in total knee arthroplasty: a double-blind, randomized controlled trial. ANZ J Surg. 2012 Jun;82(6):428–33. 21. Hasanain MS, Apostu D, Alrefaee A, Tarabichi S. Comparing the Effect of Tourniquet vs Tourniquet-Less in Simultaneous Bilateral Total Knee Arthroplasties. J Arthroplasty. 2018 Jul;33(7):2119–24. 22. Rasmussen, L. E., Holm, H. A., Kristensen, P. W., & Kjaersgaard-Andersen, P. (2018). Tourniquet time in total knee arthroplasty. *KNEE*, *25*(2), 306–313. https://doi.org/10.1016/j.knee.2018.01.002 23. Bilateral passive leg raising attenuates and delays tourniquet deflation-induced hypotension and tachycardia under spinal anaesthesia: a randomised controlled trial 24. Chesters A, Roberts I. Minimising blood loss in early trauma resuscitation. Trauma (United Kingdom). 2014;16(1):27–36. 25. Cordoba-Fernandez A, Rodriguez-Delgado FJ. Anaesthetic digital block with epinephrine vs. tourniquet in ingrown toenail surgery: a clinical trial on efficacy. J Eur Acad DERMATOLOGY Venereol. 2015 May;29(5):985–90. 26. Simon, M. A., Mass, D. P., Zarins, C. K., Bidani, N., Gudas, C. J., & Metz, C. E. (1982). The effect of a thigh tourniquet on the incidence of deep venous thrombosis after operations on the fore part of the foot. *Journal of Bone and Joint Surgery. American Volume*, *64*(2 CC-Bone, Joint and Muscle Trauma CC-Vascular), 188‐191. https://www.cochranelibrary.com/central/doi/10.1002/central/CN-00027031/full 27. Choksy SA, Lee Chong P, Smith C, Ireland M, Beard J. A randomised controlled trial of the use of a tourniquet to reduce blood loss during transtibial amputation for peripheral arterial disease. Eur J Vasc Endovasc Surg. 2006;31(6):646-50. 28. Hooper, J., Rosaeg, O. P., Krepski, B., & Johnson, D. H. (1999). Tourniquet inflation during arthroscopic knee ligament surgery does not increase postoperative pain. *Journal Canadien d’anesthesie [Canadian Journal of Anaesthesia]*, *46*(10 CC-Anaesthesia CC-Bone, Joint and Muscle Trauma CC-Pain, Palliative and Supportive Care), 925‐929. https://doi.org/10.1007/BF03013125 29. Jarrett PM, Ritchie IK, Albadran L, Glen SK, Bridges AB, Ely M. Do thigh tourniquets contribute to the formation of intra-operative venous emboli? Acta Orthop Belg. 2004;70(3):253-9. 30. Thompson SM, Middleton M, Farook M, Cameron-Smith A, Bone S, Hassan A. The effect of sterile versus non-sterile tourniquets on microbiological colonisation in lower limb surgery. Ann R Coll Surg Engl. 2011;93(8):589-90. 31. Johnson, D. S., Stewart, H., Hirst, P., & Harper, N. J. (2000). Is tourniquet use necessary for knee arthroscopy? *Arthroscopy*, *16*(6 CC-Anaesthesia CC-Bone, Joint and Muscle Trauma), 648‐651. https://doi.org/10.1053/jars.2000.4826 32. Aglietti, P., Baldini, A., Vena, L. M., Abbate, R., Fedi, S., & Falciani, M. (2000). Effect of tourniquet use on activation of coagulation in total knee replacement. *Clinical Orthopaedics and Related Research*, *371*, 169–177. http://ovidsp.ovid.com/ovidweb.cgi?T=JS&PAGE=reference&D=med4&NEWS=N&AN=10693564 33. Taft, M. Y., Kenny, E., Krehel, N., Airgood, H., Holquist, K., Gerstel, S., Corey, C., Rittenberger, J., Guyette, et al. (2015). Mitochondrial and biochemical effects of repetitive cycles of remote ischemic conditioning (RIC) in human upper and lower extremities. *Circulation*, *132*(SUPPL. 3). 34. Poggetti A, Del Chiaro A, Nicastro M, Parchi P, Piolanti N, Scaglione M. A local anesthesia without tourniquet for distal fibula hardware removal after open reduction and internal fixation: the safe use of epinephrine in the foot. A randomized clinical study. J Biol Regul Homeost Agents. 2018;32(6 Suppl. 1):57-63. |
| **Ineligible control** |
| 1. Su, J., & Cao, X. (2017). Risk factors of wound infection after open reduction and internal fixation of calcaneal fractures. *MEDICINE*, *96*(44). https://doi.org/10.1097/MD.0000000000008411 2. Bostankolu, E., Ayoglu, H., Yurtlu, S., Okyay, R. D., Erdogan, G., Deniz, Y., Hanci, V., Can, M., & Turan, I. O. (2013). Dexmedetomidine did not reduce the effects of tourniquet-induced ischemia-reperfusion injury during general anesthesia. *The Kaohsiung Journal of Medical Sciences*, *29*(2), 75–81. https://doi.org/https://dx.doi.org/10.1016/j.kjms.2012.08.013 3. Ongaya, J., Mung’ayi, V., Sharif, T., Kabugi, J., J., O., V., M., T., S., Ongaya, J., Mung’ayi, V., Sharif, T., & Kabugi, J. (2017). A randomized controlled trial to assess the effect of a ketamine infusion on tourniquet hypertension during general anaesthesia in patients undergoing upper and lower limb surgery. *African Health Sciences*, *17*(1), 122–132. https://doi.org/https://dx.doi.org/10.4314/ahs.v17i1.16 4. Whitford A, Healy M, Joshi GP, McCarroll SM, O'Brien TM. The effect of tourniquet release time on the analgesic efficacy of intraarticular morphine after arthroscopic knee surgery. Anesth Analg. 1997;84(4):791-3. 5. Jin JG, Shen HJ, Shan YL, Chen L, Zhao XY, Wang LR, et al. Effect of two administration routes of Shenmai Injection () on pulmonary gas exchange function after tourniquet-induced ischemia-reperfusion. Chin J Integr Med. 2017;23(1):18-24. 6. van der Velde J, Serfontein L, Iohom G. Reducing the potential for tourniquet-associated reperfusion injury. Eur J Emerg Med. 2013;20(6):391-6. 7. S K, Sunita. A comparative study of ketorolac vs dexmedetomidine to attenuate tourniquet induced cardiovascular response in lower limb surgery. Anaesthesia, Pain and Intensive Care. 2016;20(4):404-10. 8. Saricaoglu, F., Dal, D., Salman, A. E., Doral, M. N., Kilinç, K., & Aypar, U. (2005). Ketamine sedation during spinal anesthesia for arthroscopic knee surgery reduced the ischemia-reperfusion injury markers. *Anesthesia and Analgesia*, *101*(3 CC-Anaesthesia), 904‐9, table of contents. https://doi.org/10.1213/01.ANE.0000159377.15687.87 9. Finsen, V., & Kasseth, A. M. (1997). Tourniquets in forefoot surgery: less pain when placed at the ankle. *Journal of Bone and Joint Surgery. British Volume*, *79*(1 CC-Anaesthesia CC-Pain, Palliative and Supportive Care), 99‐101. https://doi.org/10.1302/0301-620x.79b1.7069 10. Wu, B., Xing, L., & Cao, B. (2010). [Observation of hemostatic effect of equilibrium pressure pneumatic tourniquet in internal fixation of bilateral tibia and fibula fracture]. *Zhongguo Xiu Fu Chong Jian Wai Ke Za Zhi = Zhongguo Xiufu Chongjian Waike Zazhi = Chinese Journal of Reparative and Reconstructive Surgery*, *24*(11), 1338–1340. http://ovidsp.ovid.com/ovidweb.cgi?T=JS&PAGE=reference&D=med8&NEWS=N&AN=21226357 11. Lin, L. N., Wang, L. R., Wang, W. T., Jin, L. L., Zhao, X. Y., Zheng, L. P., Jin, L. D., Jiang, L. M., & Xiong, X. Q. (2010). Ischemic preconditioning attenuates pulmonary dysfunction after unilateral thigh tourniquet-induced ischemia-reperfusion. *Anesthesia and Analgesia*, *111*(2), 539‐543. https://doi.org/10.1213/ANE.0b013e3181e368d2 12. Turan, R., Yagmurdur, H., Kavutcu, M., & Dikmen, B. (2007). Propofol and tourniquet induced ischaemia reperfusion injury in lower extremity operations. *European Journal of Anaesthesiology*, *24*(2), 185–189. http://ovidsp.ovid.com/ovidweb.cgi?T=JS&PAGE=reference&D=med6&NEWS=N&AN=16938161 13. Buciuto, R. (2014). Prospective Randomized Study of Chevron Osteotomy Versus Mitchell’s Osteotomy in Hallux Valgus. *FOOT & ANKLE INTERNATIONAL*, *35*(12), 1268–1276. https://doi.org/10.1177/1071100714550647 14. Peker K, Okesli S, Kiyici A, Deyisli C. The effects of ketamine and lidocaine on free radical production after tourniquet-induced ischemia-reperfusion injury in adults. Ulus Travma Acil Cerrahi Derg. 2019;25(2):111-7. 15. Zhuang, X.-X., Yang, L.-L., Wang, L., Mo, Y.-C., Pan, Y.-Y., Dai, Q.-X., Lu, Y., & Wang, J.-L. (2019). [Effect of transcutaneous electrical acupoint stimulation on pulmonary function and oxidative stress response in lower extremity surgery patients using tourniquet]. *Zhen Ci Yan Jiu = Acupuncture Research*, *44*(8), 594–598. https://doi.org/https://dx.doi.org/10.13702/j.1000-0607.180709 16. Stimpson J, Gill DF, Memarzadeh A, Dunne M, Perry L, Magan A, et al. Reducing the Hypertensive Effects of the Prolonged Surgical Tourniquet Using a Dual-Cuff Strategy: a Prospective Randomized Controlled Trial. J foot ankle Surg. 2019;58(6):1177‐1186. |
| **Tourniquet use unspecified** |
| 1. Moo IH, Chen JYQ, Pagkaliwaga EH, Tan SW, Poon KB. Bone Wax Is Effective in Reducing Blood Loss After Total Knee Arthroplasty. J Arthroplasty. 2017 May;32(5):1483–7. 2. Paiement GD, Renaud E, Dagenais G, Gosselin RA. Double-blind randomized prospective study of the efficacy of antibiotic prophylaxis for open reduction and internal fixation of closed ankle fractures. J Orthop Trauma. 1994;8(1):64-6. 3. Svatos F, Bartoska R, Skala-Rosenbaum J, Dousa P, Pacovsky V, Krbec M. [Calcaneal fractures treated by open reduction and internal fixation with a locking compression plate (LCP). A prospective study. part I: basic analysis of the group]. Acta Chir Orthop Traumatol Cech. 2011;78(2):136-30. 4. Mas, E., Barden, A. E., Corcoran, T. B., Phillips, M., Roberts, L. J., & Mori, T. A. (2011). Effects of spinal or general anesthesia on F₂-isoprostanes and isofurans during ischemia/reperfusion of the leg in patients undergoing knee replacement surgery. *Free Radical Biology & Medicine*, *50*(9), 1171‐1176. https://doi.org/10.1016/j.freeradbiomed.2011.01.021 5. Ding, L., He, Z., Xiao, H., Chai, L., & Xue, F. (2013). Risk Factors for Postoperative Wound Complications of Calcaneal Fractures Following Plate Fixation. *FOOT & ANKLE INTERNATIONAL*, *34*(9), 1238–1244. https://doi.org/10.1177/1071100713484718 6. d'Heurle A, Kazemi N, Connelly C, Wyrick JD, Archdeacon MT, Le TT. Prospective Randomized Comparison of Locked Plates Versus Nonlocked Plates for the Treatment of High-Energy Pilon Fractures. J Orthop Trauma. 2015;29(9):420-3. 7. Saied, A., Ostovar, M., Mousavi, A. A., & Arabnejhad, F. (2016). Comparison of intramedullary nail and plating in treatment of diaphyseal tibial fractures with intact fibulae: A randomized controlled trial. *INDIAN JOURNAL OF ORTHOPAEDICS*, *50*(3), 277–282. https://doi.org/10.4103/0019-5413.181793 |
| **Systematic review/meta-analysis** |
| 1. Smith TO, Hing CB. The efficacy of the tourniquet in foot and ankle surgery? A systematic review and meta-analysis. Foot Ankle Surg. 2010;16(1):3-8. 2. Praestegaard M, Beisvag E, Erichsen JL, Brix M, Viberg B. Tourniquet use in lower limb fracture surgery: a systematic review and meta-analysis. European journal of orthopaedic surgery & traumatology : orthopedie traumatologie. 2019;29(1):175-81. 3. Jiang X, Yu B, Qu W, He J. RETRACTED: Meta-analysis on the efficacy of tourniquet on ankle trauma surgery(Retracted article. See vol. 20, 34, 2015). EUROPEAN JOURNAL OF MEDICAL RESEARCH. 2013;18. |
| **Study relates to upper limb** |
| 1. Omeroglu H, Ucaner A, Tabak AY, Guney O, Bicimoglu A, Gunel U. The effect of using a tourniquet on the intensity of postoperative pain in forearm fractures. A randomized study in 32 surgically treated patients. Int Orthop. 1998;22(6):369-73. |
| **Not accessible/not in English** |
| 1. Assessment of the Use of Tourniquet in the Plating of Non-Union of the Tibia by A Ajibade, AM Oluwasina, FO Awonusi in Nigerian journal of orthopaedics and trauma; Vol. 12 No. 1 (2013); published 2014 2. Xie C-Y, Xiao J-F, Zhao Z-L. [Protective Effect of Ulinastatin against Activation of Tourniquet-Induced Platelet Mitochondria Apoptotic Signaling]. Zhongguo shi yan xue ye xue za zhi. 2015;23(4):1087–91. |

Supplementary Table 2: List of excluded studies along with reasons for exclusion.
